# Origin of the Time Lag Phenomenon and the Global Signal in Resting-State fMRI

**DOI:** 10.3389/fnins.2020.596084

**Published:** 2020-10-29

**Authors:** Shiori Amemiya, Hidemasa Takao, Osamu Abe

**Affiliations:** Department of Radiology, Graduate School of Medicine, The University of Tokyo, Tokyo, Japan

**Keywords:** BOLD fMRI, autoregulation, neurovascular coupling, spatiotemporal dynamics, hemodynamics, global signal

## Abstract

The global mean signal of resting-state fMRI (rs-fMRI) shows a characteristic spatiotemporal pattern that is closely related to the pattern of vascular perfusion. Although being increasingly adopted in the mapping of the flow of neural activity, the mechanism that gives rise to the BOLD signal time lag remains controversial. In the present study, we compared the time lag of the global mean signal with those of the local network components obtained by applying temporal independent component analysis to the resting-state fMRI data, as well as by using simultaneous wide-field visual stimulation, and demonstrated that the time lag patterns are highly similar across all types of data. These results suggest that the time lag of the rs-fMRI signal reflects the local variance of the hemodynamic responses rather than the arrival or transit time of the stimulus, whether the trigger is neuronal or non-neuronal in origin as long as it is mediated by local hemodynamic responses. Examinations of the internal carotid artery signal further confirmed that the arterial signal is tightly inversely coupled with the global mean signal in accordance with previous studies, presumably reflecting the blood flow or blood pressure changes that are occurring almost simultaneously in the internal carotid artery and the cerebral pial/capillary arteries, within the low-frequency component in human rs-fMRI.

## Introduction

The advent of resting-state functional magnetic resonance imaging (rs-fMRI) has brought a means of investigating spontaneous neural activity and its macroscopic organization characterized by the coherence of the activity. The spatial patterns identified as areas with synchronous oscillation of the blood oxygenation level-dependent (BOLD) signal are termed resting-state networks (RSNs) (Fox et al., 2005). These networks are closely related to the anatomical connectivity among the neural subsystems that have been revealed by a wide variety of visual, sensorimotor, and cognitive task paradigms (Vincent et al., 2007; Zhang et al., 2010). However, the neurophysiology of the phenomenon, or the mechanism that controls and coordinates the intrinsic synchronization within and across neural systems, largely remains to be established (Drew et al., 2020). Undoubtedly, the successful mapping of the flow of information among the population of neurons would be the key to understanding all these and other enduring questions in neuroscience. Against this backdrop, attempts have been made to map the flow of neural signals by analyzing the spatiotemporal patterns of the rs-fMRI BOLD signal (Majeed et al., 2009, 2011; Mitra et al., 2014, 2015; Amemiya et al., 2016, 2019; Takeda et al., 2016; Belloy et al., 2018; Abbas et al., 2019; Raut et al., 2019).

Several animal studies have reported the existence of the global propagation of the intrinsic neural activities (Stroh et al., 2013; Matsui et al., 2016; Vanni et al., 2017) and that the time lag of the BOLD signal could correspond to such electrophysiological infra-slow activity (Matsui et al., 2016). However, such findings were not necessarily consistent with the time lag phenomenon in human fMRI studies. In the studies exploring the existence of the global signals of neural origin, rs-fMRI time lag structures were found to be multi-dimensional (Mitra et al., 2014, 2015; Amemiya et al., 2016). However, such global signals were not identified even when a temporal independent component analysis (ICA) was applied to allow detection of the overlapping activities (Amemiya et al., 2019)—contradicting the assumptions that there are multiple paths of the infra-slow neural activities independent of the underlying vascular dynamics.

BOLD fMRI is an indirect measurement of the neural activity exploiting a vascular response that is necessarily influenced by vascular dynamics. Accordingly, the validity of the inferences is crucially dependent on accurate estimates of the hemodynamic responses. The assumption that global mean signal (GMS) regression can eliminate the underlying vascular effect might lack adequate empirical support or conceptual plausibility. Therefore, not only from a physiological point of view, it is of practical importance in neuroimaging studies to understand the mechanism that gives rise to the global signals and their time lag, regardless of their origin.

Several lines of evidence to date indicate the contribution of physiological noise to the GMS (Liu T.T. et al., 2017). Interestingly, the GMS exhibits a constant time lag, as if it were traveling through the cerebrovascular system. In a series of studies on low-frequency oscillations of the BOLD signal, Tong et al. demonstrated that the signal measured in the peripheral vasculature using near-infrared spectroscopy is well correlated with the signal from the cerebrovascular system (Tong and Frederick, 2010, 2012, 2014a,b; Tong et al., 2013; Tong and Hocke, 2014). Furthermore, independent clinical studies measuring the rs-fMRI signal time lag using GMS as the reference signal have consistently identified rs-fMRI signal delay under hypoperfusion or ischemia comparable to that in dynamic susceptibility contrast (DSC) perfusion imaging (Lv et al., 2013; Amemiya et al., 2014; Christen et al., 2015). The delay was even evident in MR-defined ischemic penumbra or stroke core (Lv et al., 2013; Amemiya et al., 2014), where normal spontaneous neuronal activity is known to be absent, supporting the view that the major source of the GMS and its time lag are vascular in origin.

Under physiological conditions, the local vascular mean transit time is almost homogenous across the cerebral cortex and prolongs monotonically in proportion to the cerebral perfusion pressure decrease (Sette et al., 1989; Ito et al., 2003; Ibaraki et al., 2007). Consequently, the arrival and peak time of the venous flow are generally dependent on those of the arterial flow. That is, if the source of the signal simply flows through the vessels from the arteries to veins like a contrast agent, as in DSC perfusion imaging, the BOLD signal time lag would also reflect the arterial arrival and peak time difference, which is up to 500 ms (Hendrikse et al., 2008; MacIntosh et al., 2010), though the peak time difference would be amplified in the veins.

Alternatively, the GMS time lag might mainly reflect the variance in local hemodynamic responses. Major candidate sources of the GMS, such as the cardiac and respiratory factors, are known to cause local hemodynamic responses that are similar to neurovascular coupling, as is seen in the experimental manipulation of these factors (Battisti-Charbonney et al., 2011). For example, vasodilation in response to arterial partial pressure of carbon dioxide mainly occurs at the arterioles and precapillary sphincter (Ainslie and Duffin, 2009). Therefore, if it is the factor that triggers the GMS, in addition to the traveling time difference of the carbon dioxide, we need to take into account the local variance of the hemodynamic responses.

In this study we hypothesized that the BOLD time lag phenomenon is related to the variance of the local hemodynamic responses, which can vary on the order of seconds, rather than the time lag on the part of the stimulus or trigger, whether it is neuronal or non-neuronal in origin. Indeed, a similar pattern of the time lag has been shown in studies examining the BOLD signal changes associated with end-tidal carbon dioxide or with a breath-holding challenge (Chang and Glover, 2009). Although the time lag is less conspicuous, a similar contrast between the primary and association cortices has also been demonstrated in the temporal parameters of the hemodynamic response function (HRF) in task fMRI (Taylor et al., 2018). Therefore, it seems possible that the time lag phenomenon is caused by a *substantially* synchronous stimulus producing time lag depending on the local arterial density, or more precisely, the time required to increase the local blood flow, which is also dependent on the status of the arterioles of capillaries in terms of the vascular reactivity in each region (Roc et al., 2006; Bonakdarpour et al., 2007; Amemiya et al., 2012). Once the delay is embedded in its signal, the venous blood further drains into larger veins and sinuses carrying the BOLD signal as it is just like the contrast agent. The term *substantial* is used here to allow for the possibly traveling stimulus on the order of milliseconds, which is unlikely to be reflected in the time lag in this scenario. Another point to note is that BOLD signal time lag measurement is unlikely to distinguish the traveling time of a stimulus if carried out along with the blood flow before the induction of hemodynamic response time lags in the local veins because the two measures are likely proportionate to each other.

To test the hypothesis, we first examined the presence of the time lag phenomenon in the rs-fMRI local components obtained by applying the temporal ICA to the Human Connectome Project (HCP) rs-fMRI datasets (Amemiya et al., 2019). As previously well-explored in Smith et al. (2012), these components could subdivide and/or reorganize the conventional RSNs. Nevertheless, they are similar to the conventional RSNs in that they are confined within the functionally relevant cerebral structures as opposed to the GMS, which is almost ubiquitous in the systemic vasculatures (Tong and Frederick, 2010; Tong et al., 2012; Li et al., 2018). If the GMS time lag is the reflection of the “traveling signal,” while the local network activities are synchronous and non-traveling as measured using magnetoencephalography (Brookes et al., 2011) or electroencephalography (Liu Q. et al., 2017), a similar time lag would not be found for the local components. In contrast, if the time lag is produced by the variance in the vascularity, resulting in the varied HRF, a similar time lag would also be found for the synchronized network signals.

The investigation of the resting-state local signal time lag might be more beneficial in terms of rs-fMRI methodology. However, the problem is that many independent components (ICs) are confined within areas with a similar global signal time lag, limiting the verification of our hypothesis. The overall ambiguity of the source of signals in rs-fMRI could be another problem. Therefore, in our second experiment, we also performed the task fMRI to further compare the time lag of the signals resulting from neural activation with that of the resting-state GMS. For this purpose, we prepared a projection system that enables the wide-field visual stimulation to activate the whole visual cortex simultaneously so that the findings would be more conclusive. Lastly, to address the question regarding the origin of the GMS, we examined the internal carotid artery signal using the rs-fMRI data acquired in Experiment 2. In addition to the time lag analysis following the procedure in previous studies (Tong et al., 2019b; Yao et al., 2019), we included some evaluation of the characteristics of the arterial signal to further clarify the origin of the phenomenon.

## Materials and Methods

### Experiment 1. Resting-State Functional Networks’ Time Lag Mapping

#### Data

The data are originally from the WU-Minn HCP young healthy adults (ages 22–35) S1200 release that provides a paired dataset of the same group of subjects (day 1 and day 2). We used the same dataset previously analyzed with temporal ICA to examine the global and local signal contributions to the RSN synchronization (Amemiya et al., 2019). Briefly, the data of 50 subjects who underwent 3 T resting-state fMRI sessions without quality control issues, and whose mean framewise displacement was less than 0.2 mm, were included in the analysis. All preprocessing and data analyses were performed for each dataset, respectively, in the same way.

#### fMRI Data Analysis

HCP imaging and pre-processing protocol have been previously described in detail (Glasser et al., 2013; Smith et al., 2013; Van Essen et al., 2013; Griffanti et al., 2014; Salimi-Khorshidi et al., 2014). Further pre-processing and analysis of the data were performed using tools from the AFNI libraries and in-house scripts written and implemented in MATLAB 9.3 and 9.7 (MathWorks, Natick, MA, United States), as is described elsewhere (Amemiya et al., 2019). In short, linear trends were removed from the HCP data that had been processed with subject-level ICA noise reduction (sICA + FIX), and the data were band-pass filtered at 0.01–0.1 Hz. The pre-processed data were temporally concatenated across runs to create a single 4D dataset of 120,000 time points (1,200 frames × 50 subjects × 2 phase-encoding directions) for test (Dataset 1) and re-test (Dataset 2) dataset, respectively. For temporal ICA, we employed a strategy adapted from Smith et al. (2012) to perform group-wise spatial ICA in advance of the final temporal ICA using FastICA (Hyvärinen, 1999). The temporal ICA decomposition was based on the Icasso algorithm (Himberg et al., 2004) that estimates the most appropriate decomposition yielding a set of reproducible IC clusters, which found 28 and 30 components for each dataset, respectively.

For all temporal ICs, the time-series of each run, once concatenated to be subjected to a temporal ICA, were de-concatenated so that the following analyses could be performed for each run separately. For each IC, the time lag map was computed as the relative time lag *t* that gives the best positive fit between each voxel’s time-series and the time-shifted (±5.8 s or ±8 TR) IC time-series using cross-correlation analysis (Supplementary Figure 1). All other types of time lag maps in Experiments 1 and 2 were similarly computed by using either the GMS or the task-induced BOLD signal as the reference signal. All data were up-sampled to a resolution of 0.18 s (1/4 TR) for the analysis (Tong et al., 2017, 2019b; Yao et al., 2019). The magnitude map of each IC was then computed as the Pearson’s correlation coefficients between each voxel’s time-series and the IC time-series that was shifted as much as *t*. Based on the spatial pattern, 21 and 20 ICs, respectively, were identified as local functional network ICs rather than global signal components (Amemiya et al., 2019). The GMS was computed as the average signal within a gray matter mask that was created by thresholding Montreal Neurological Institute (MNI) template at 30% or larger probability of being gray matter.

#### Time Lag Comparison

To examine the hypothesis that a similar time lag phenomenon is found in the local ICs, the zero-centered (by linear detrending) relative time lag of each IC signal was compared with that of the GMS voxel-wisely for each subject’s individual run. For visualization and superimposition of the time lag maps, each IC time lag was adjusted so that the mean of the IC time lag range equals that of the GMS for each subject’s data. To minimize the bias in the time lag estimation for each signal, the time-series correlation threshold was set at the Pearson’s correlation coefficient of *r* > 0.3 (Tong et al., 2017, 2019b; Yao et al., 2019), which overall corresponded to the adjusted Z-score of >3 (*p* < 0.0027) computed by the xDF approach with the “adaptive truncation” methods for the regularization of the autocorrelation function (Afyouni et al., 2019). All the ICs whose average magnitude map had voxels that consistently survived the time-series correlation threshold across 100 runs were included in the analysis. The correlation between the two time lag sets that was computed as Pearson’s correlation coefficient for each run was Fisher’s Z transformed and tested by using a two-tailed *t*-test over runs against the null hypothesis of no correlation.

### Experiment 2. Visual Task and Resting-State fMRI

#### Participants

Nine healthy subjects (four men and five women; 32.5 ± 5.6 years of age), who gave written informed consent, participated in the study. All were free of abnormal neurological history, taking no medication and with normal or corrected-to-normal visual acuity. None showed any abnormality of the brain on an MRI. All procedures complied with the Declaration of Helsinki, and the Institutional Review Board of the University of Tokyo approved the study.

#### Visual Stimulus

The visual stimulus was back-projected using a video projector and a mirror (300 mm × 300 mm) onto a rear-screen (300 mm × 200 mm) that was set 12 cm above the subject, subtending a horizontal and vertical visual angle of 138 and 118 degrees, respectively. The participants viewed the screen from inside the head coil through glasses with a power of +6.0 diopters (MediGlasses, Cambridge Research Systems, Kent, United Kingdom) to focus on the stimulus. The projection system was made entirely of plastic and prepared according to Greco et al. (2016). The stimulus was based on a black and white checkerboard pattern comprising 24 segments and 26 rings, with the pattern reversed polarity at 6 Hz. The stimulus was presented in a block design comprising periods of 12 s of activation and 36 s of controls repeated for six cycles (task 1) following 11 s of pre-scan and 6 s of control condition. For task 2, the same stimulus was presented for 6 s, followed by 24 s of control, and repeated for ten cycles. During the control phase, a gray screen with the luminance equal to the average of the black and white checks was presented. The subjects were told to gaze at a central red fixation dot throughout the runs. Immediately after each run, the subjects were asked to wave their hands and to report wakefulness during the scan subjectively.

#### Scanning

For each subject, 6 (or 10 in two subjects) runs of visual task fMRI (3 runs of task 1 and task 2, respectively), a single run of rs-fMRI, and high spatial resolution T1-weighted (MPRAGE) and T2-weighted imaging were performed with a whole-body 3.0T MR unit (MAGNETOM Skyra, Siemens Healthcare, Erlangen, Germany) using a 32-channel phased-array head coil. For the fMRI scans, we used a multiband echo-planar imaging (EPI) sequence employing a simultaneous multi-slice imaging technique (Breuer et al., 2006; Moeller et al., 2010; Setsompop et al., 2012; Cauley et al., 2014) (Multi-Band EPI Package, Release 016a VE11C, Center for Magnetic Resonance Research, University of Minnesota)^1^. The acquisition parameters were as follows: TR = 750 ms; TE = 32 ms; flip angle = 62°; matrix size = 70 × 70; FOV = 210 mm × 210 mm with 100% phase sampling and no interpolation; 3 mm-thick sections with no gap; 60 sections; 400/392/408 frames (resting/task1/task2 fMRI); multiband factor 4; iPAT factor 2; blipped controlled aliasing in parallel imaging results in higher acceleration (CAIPIRINHA) phase shift of FOV/4; and a leakage block technique (Cauley et al., 2014) was used for reconstruction. A total of 3 and 7 runs were excluded from the analysis for task 1 and task 2, respectively, because of non-compliance with the instructions.

#### Data Analysis

##### Preprocessing

All task and rs-fMRI data were processed using SPM12 (Wellcome Department of Cognitive Neurology, London, United Kingdom) implemented in MATLAB 9.7 (MathWorks, Inc., Natick, MA, United States). Data were corrected for differences in acquisition time between slices, realigned to the first volume to account for movement artifacts, spatially aligned with anatomic T1-weighted images, and then normalized to the MNI space via the unified segmentation approach (Ashburner and Friston, 2005) using the T1-weighted images and spatially smoothed with a Gaussian kernel with 8 mm full-width at half-maximum.

##### Visual activation area

For task fMRI data, statistical analysis was performed in two stages in a mixed-effects model. In the first-level analysis, the visual task condition was defined. The canonical HRF was convolved with a sequence of delta functions to form covariates for the general linear model. Six motion parameters were also included as nuisance regressors in the model, and the data were high-pass filtered at 1/128 Hz. In the second-level analysis, statistical parametric maps of the task condition from each run were tested with a *t*-test to obtain group-level activation maps. Since the activation patterns of task 1 and task 2 were similar, group analysis was performed combining the two first-level analysis results (number of runs: 28 + 24 = 52). A mask of activation areas was made by including the voxels showing significant positive activation at a voxel-wise familywise error rate corrected threshold of *p* < 0.001 with a cluster size of >10 voxels.

##### Time lag comparison within the visual areas

For each run of task and rs-fMRI, the time lag map was computed as described in Experiment 1 by using the averaged time-series within the mask as the reference signal for the task fMRI data and the GMS for the rs-fMRI data, respectively. The correlation between the two time lag sets that was computed as Pearson’s correlation coefficient for each run was Fisher’s Z transformed and tested by using a two-tailed *t*-test over runs against the null hypothesis of no correlation.

##### The internal carotid artery signal

The time lag of the arterial signal was also measured in the extracranial internal carotid arteries following the procedure described in Tong et al. (2019b) and Yao et al. (2019). The regions of interest (ROIs) were semi-automatically obtained by setting an intensity threshold within the areas surrounding each carotid artery using MPRAGE T1-weighted images, in which large arteries show high signal intensity due to the time-of-flight in-flow effect. In addition to the time lag analysis, we compared the arterial signal with the signal of the surrounding tissue to further clarify the origin of the signal change. For each arterial ROI, a peripheral ROI of the surrounding tissues was created by inflating the arterial ROI that was subsequently subtracted by the original ROI. All ROIs were visually inspected, and contaminated vessels were carefully removed. The arterial and peripheral signals were measured using the non-warped and non-smoothed data. The average signal of the arterial and peripheral ROIs was measured for each internal carotid artery and compared using a paired *t*-test. Cross-correlation analysis was performed as described above to measure the time lag between the arterial signal (right and left carotid arteries) and the GMS for each subject.

## Results

### Experiment 1. Time Lag Map of the Resting-State Functional Networks (HCP Data)

**FIGURE 1:**
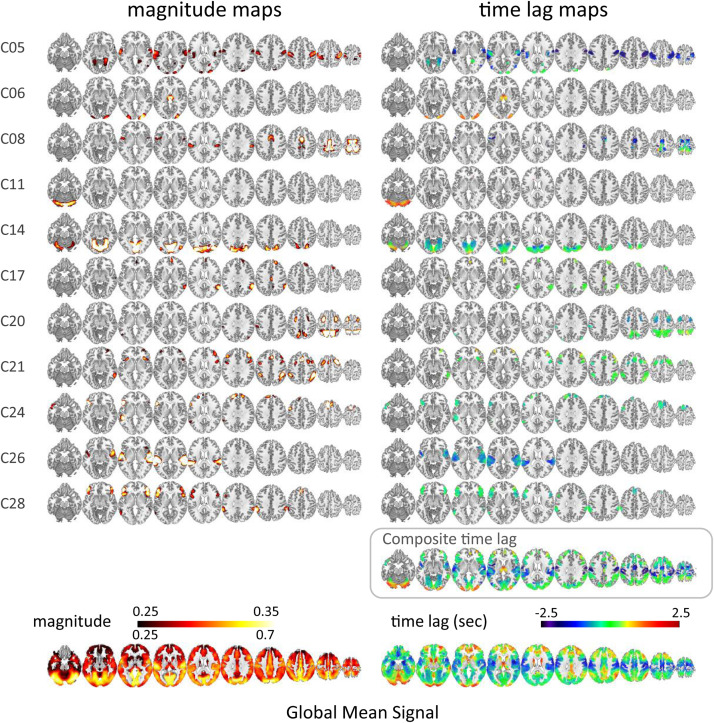
Time lag map of the resting-state functional networks (HCP data, Dataset 1). The magnitude (time-series correlation) and the time lag maps of the 10 ICs from Dataset 1, which was judged as a local component in the previous study (Amemiya et al., 2019) and that survived the time-series correlation threshold of Pearson’s *r* > 0.3 across the 100 runs are shown in the left and the right column, respectively. The bottom row is the magnitude and time lag maps of the global mean signal. A larger time lag correlation was seen for the ICs with larger areas of high magnitude, such as the sensorimotor cortex (C05) or the visual cortex (C14). C05 is the component originally judged as a global component whose time lag pattern was not significantly correlated with that of the global mean signal. The composite map incorporating all IC lag maps shows a marked similarity between the time lag maps of the local network signals and that of the global mean signal (*r* = 0.82, *p* < 2.2 × 10^–308^).

**FIGURE 2:**
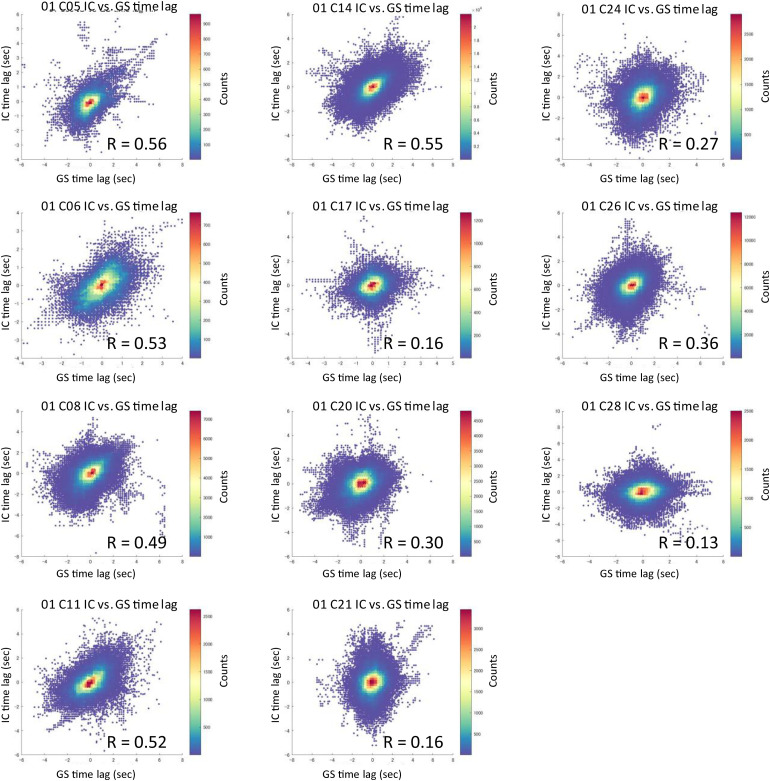
2D histogram of the pooled time lag data (HCP data, Dataset 1). For each IC, a 2D histogram shows the relationship between the IC vs. global mean signal time lag (pooled data across subjects). All IC time lags were significantly correlated with that of the global mean signal when compared within the areas surviving the time-series correlation threshold of Pearson’s *r* > 0.3 (*r* = 0.37 ± 0.17, *p* < 0.001) (GMS = global mean signal).

Of the 21 and 20 resting-state functional network ICs identified as the local components by applying temporal ICA to HCP datasets (Amemiya et al., 2019), 10 and 11 ICs, respectively, that survived the time-series correlation threshold of Pearson’s *r* > 0.3 across the 100 runs were included in the analysis. One IC that was judged as being a global component based on the spatial pattern in the previous study (Amemiya et al., 2019) but whose time lag was not significantly correlated with that of the GMS was also included in the analysis (Dataset 1, C05). The magnitude and time lag maps of the 11 ICs, and those of the GMS, are shown in Figure 1 and Supplementary Figure 2, respectively. All IC time lags were significantly correlated with that of the GMS (*r* = 0.37 ± 0.17, *p* < 0.001; re-test, *r* = 0.31 ± 0.13, *p* < 0.001; Figure 2 and Supplementary Figure 3, 2D histogram of the pooled individual data). A larger time lag correlation was seen for the ICs with larger areas of high magnitude, such as the sensorimotor (C05 in Dataset 1) or the visual cortex (Figure 1, C14 in Dataset 1; Supplementary Figure 2, C16 and C21 in Dataset 2). A composite map, incorporating all functional network (local IC) time lag maps for each dataset shows a marked similarity between the time lag maps of the functional network signals and that of the GMS (Dataset 1, *r* = 0.82, *p* < 2.2 × 10^–308^; Dataset 2, *r* = 0.86, *p* < 2.2 × 10^–308^) (Figure 1 and Supplementary Figure 2). A larger time lag between the IC and GMS time lag was more likely to be found at the periphery of each region (Supplementary Figure 4).

### Experiment 2. Visual Task and Resting-State fMRI

#### Time Lag Map of the Visual Task fMRI

**FIGURE 3:**
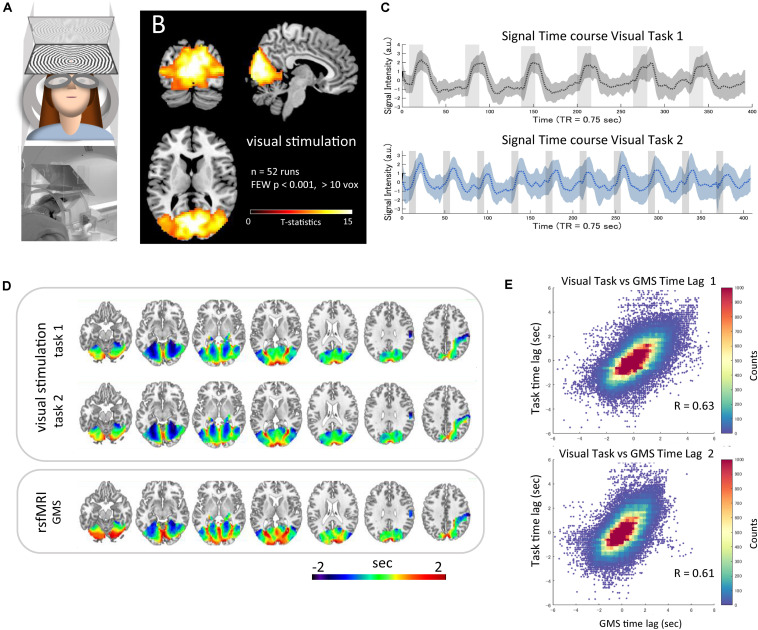
Time lag of visual stimulation data. The wide-field visual stimulation system **(A)** activated the whole visual cortex from the primary to the high-order areas, including the hMT/V5 as well as the lateral geniculate bodies [**(B)** t-map, familywise error-corrected *p* < 0.001]. The mean time-series of each task averaged within the mask across all runs are plotted as a dotted line with SD indicated as shading **(C)**. The average time lag maps obtained by using the mean time-series as the reference signals are shown in panel **(D)**. These time lag maps of the BOLD response to the simultaneous neural activation were highly correlated with the time lag map of the resting-state global mean signal (task 1, *r* = 0.64, *p* < 9.5 × 10^–19^; task 2, *r* = 0. 61, *p* < 3.4 × 10^–13^) [**(E)** 2D histogram of the pooled time lag data across subjects].

The wide-field visual stimulation system (Figure 3A) activated the whole visual cortex from the primary to the high-order areas, including the hMT/V5 near the ascending limb of the inferior temporal sulcus (Dumoulin et al., 2000) as well as the lateral geniculate bodies. The statistical map of t-values thresholded at familywise error-corrected *p* < 0.001 is visualized in Figure 3B. The mean time-series of each task averaged within the mask across all runs are plotted with the standard deviation (SD) (Figure 3C). The average time lag maps obtained with the time-series as the reference signals are shown in Figure 3D. These time lag maps of the BOLD response to the simultaneous neural activation were not only similar to each other (*r* = 0.95, *p* < 2.2 × 10^–308^) but also highly correlated with that of the resting-state fMRI GMS (task 1, *r* = 0.64, *p* < 9.5 × 10^–19^; task 2, *r* = 0. 61, *p* < 3.4 × 10^–13^) (Figures 3D,E). The two time lag measures showed high correlation even at the subject level (task 1 vs. GMS, *r* = 0.64 ± 0.11, range 0.47–0.88; task 2 vs. GMS, *r* = 0.64 ± 0.14, range 0.39–0.92). The time lag maps of the GMS obtained from the nine scans were similar to those of the HCP datasets (*r* = 0.60 and 0.61 for Dataset 1 and Dataset 2, respectively). Each voxel’s signal time-series was highly correlated with the reference time-series, and the strength of the correlation was not significantly correlated with the absolute time lag (task 1, *r* = −0.08, *p* < 0.10; task 2, *r* = −0. 05, *p* < 0.20).

The average frame-wise displacements of the task 1 (28 runs), task 2 (24 runs), and resting-state fMRI scans (9 runs) were 0.10 ± 0.02, 0.10 ± 0.02, and 0.09 ± 0.02 mm, respectively.

#### Experimental Arterial Signal

**FIGURE 4:**
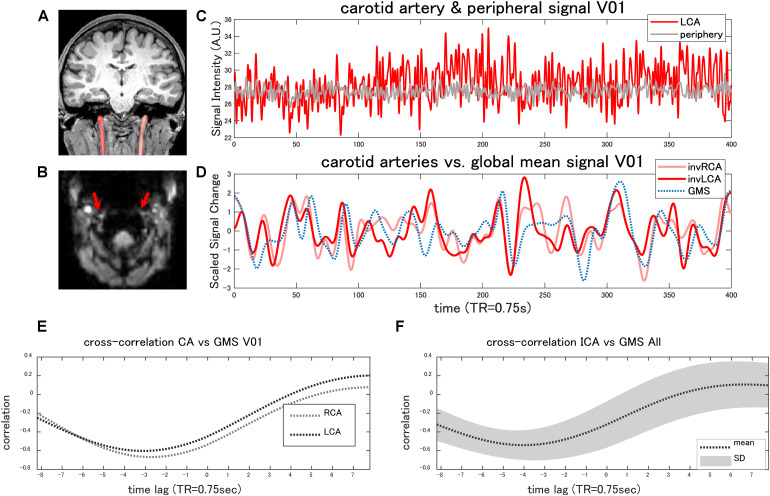
Internal carotid artery signal. The coronal T1-weighted image (MPRAGE) shows the ROI of the bilateral internal carotid arteries (CAs) of a representative case **(A)**. The original EPI image shows high signal intensity in the external carotid arteries or in the vertebral arteries **(B)**. Although the internal carotid arteries (red arrows) were less conspicuous **(B)**, the average signal within the internal carotid artery was higher compared with the peripheral tissues **(C)** in the majority of the cases (paired *t*-test, *p* < 0.0073). Panel **(D)** shows the time-series of the global mean signal (GMS) and those of the internal carotid arteries of the same case. The cross-correlation analysis found that the global mean signal of the rs-fMRI was negatively correlated (*r* < −0.3) with the arterial signal **(E)** in the majority of the cases, with an average time lag of −2.7 ± 1.36 s. The average cross-correlation with the standard deviation (SD) is plotted in panel **(F)**. The arterial signals are inversed in panel **(D)** to facilitate the comparison (invRCA and LCA = inversed right and left carotid artery).

Iinternal carotid artery ROIs of a representative case are shown in Figure 4A. The original EPI images showed high signal intensity in the external carotid arteries or the vertebral arteries in all the subjects (Figure 4B). Although the internal carotid artery was less conspicuous, presumably due to the susceptibility artifact caused by the inhomogeneity of the local magnetic field near the pharynx or by the higher flow, the average signal in the internal carotid artery was, on the whole, significantly higher compared with the peripheral tissues (paired *t*-test, *p* < 0.0073) (Figure 4C).

The cross-correlation analysis found that the GMS of the rs-fMRI was negatively correlated (*r* < −0.3) with the arterial signal in 15 of 18 carotid arteries (*r* = −0.57 ± 0.13) (Figures 4D,E), with an average time lag of −2.7 ± 1.36 s. In the other three carotid arteries, the maximum correlation ranged from −0.19 to 0.58, with a delay of 2.1–5.4 s. All of these arteries showed a lower signal to the periphery. The cross-correlation plot combining the all subject data shows a negative peak at 4 s (Figure 4F).

## Discussion

By examining the time lag of the resting-state functional network signals, we demonstrated that their spatiotemporal pattern is similar to that of the GMS. Given that the functional network components are locally confined and non-globally traveling signals, reflecting the synchronous activities as measured using magnetoencephalography (Brookes et al., 2011) or electroencephalography (Liu Q. et al., 2017), their time lag is likely to reflect the local variance of the hemodynamic responses. This view was further confirmed more directly in Experiment 2, in which we showed that simultaneous visual stimulation results in a similar time lag within the visual cortex. Since differences in the response timing of the visual neurons to varied types of stimulation are less than 100 ms across the mammalian visual system (Nowak and Bullier, 1997; Bair et al., 2002), neural factors are less likely to cause time lag on the order of seconds in the HRF. Such a view is also supported by empirical data showing that the HRF onset or peak time, known to vary up to several seconds (Taylor et al., 2018), does not differ so much depending on their “functional” anatomy (Puckett et al., 2014).

These results suggest that the time lag of the rs-fMRI signal reflects the local variance of the hemodynamic responses rather than the arrival or transit times of the stimulus, which is in contrast to the general assumptions made in previous studies.

The local hemodynamic response to neural stimulation measured as BOLD signal change is sensitive to the vascularity in each region. It is delayed in the areas supplied by the small (collateral) arteries that are often found distal to the obstructed or narrowed arteries in ischemic patients (Roc et al., 2006; Bonakdarpour et al., 2007; Amemiya et al., 2012). In healthy subjects, the areas showing the BOLD signal delay seem to correspond well to the watershed areas. Located at the borders of the perfusion territories, these areas supplied by the most distal and smallest branches of the major arteries are known as the regions most vulnerable to a sudden drop in blood pressure or proximal occlusion of the major arteries (Wodarz, 1980; Zülch, 1985; Hossmann and Heiss, 2016). In the frontal lobes, the areas extending from the anterior horn of the lateral ventricle to the cortex showing delayed BOLD signal (Figure 1 and Supplementary Figure 2) correspond to the anterior watershed areas between the anterior and middle cerebral artery territories. On the other hand, the parieto-temporo-occipital wedge-shaped-delayed regions extending from the occipital horn of the lateral ventricle to the cortex (Figure 1 and Supplementary Figure 2) correspond to posterior watershed areas (Wodarz, 1980; Zülch, 1985; Hossmann and Heiss, 2016). Importantly, given that the BOLD response is dependent on the local hemodynamics, it is considered the local vascularity rather than the geographical localization of each region that matters. That is, the reason for the delayed BOLD signal in the association cortex seems not only because the arrival time of the blood from the heart is longer but also because the vascularity or the arteriolar/capillary density is lower in the higher-order regions compared with the primary cortices (Harrison et al., 2002; Weber et al., 2008).

These observations offer some insights into the origin of the GMS. Given that the source of the signal can be substantially synchronous across the cerebrovascular system or their traveling time lag is blood flow perfusion dependent, most cardiac and respiration related factors are the likely candidates of the phenomenon. In humans, oscillations in heart rate and blood pressure represent both autonomic neural fluctuations and mechanically induced central blood volume changes in synchrony with respiration (Cohen and Taylor, 2002). The interrelated factors, namely, the variations of the heart rate (Chang et al., 2009) and the blood pressure (Zhang et al., 2000), respiration volume per time (Birn et al., 2006, 2008) and partial pressure of end-tidal carbon dioxide (Wise et al., 2004) are indeed known to alter the cerebral blood flow or blood volume, thereby affecting the BOLD signal in the low-frequency range of interest. Importantly, all these factors are considered to affect the cerebrovascular system substantially simultaneously (or with a perfusion-dependent traveling time lag in case of carbon dioxide). The abrupt change in blood pressure is associated with the immediate change in the blood flow, with the time lag between the arterial and the venous tracing of 0.2 s and with a regulatory reaction measured as the flow velocity beginning at 0.95 and 2.6 s for the middle cerebral artery and the straight sinus, respectively (Aaslid et al., 1991). The findings suggest that the vessels are stiff enough to immediately convey the pressure change to the periphery but that the changes in the flow associated with the hemodynamic response may take several seconds to be observed in the BOLD signal (note that the propagation of flow changes and transit time of the blood are different measures).

The physiological changes, even being synchronously induced in the capillary or pial arterioles just as in the case of the synchronous neural activation, would cause the local cerebral blood flow change, with a varied time lag depending on the arterial density in each region. The changes in the blood flow would be reflected, almost as they are, in the BOLD signal changes in the local veins given that the (spontaneous) BOLD signal is known to be significantly synchronized with the cerebral blood flow fluctuations in most cortical areas (Tak et al., 2014, 2015; Chen et al., 2015; Chiacchiaretta et al., 2018). Consistent with the hypothesis, direct observation of the low-frequency hemodynamic oscillations in mice using optical intrinsic signal imaging showed global fluctuations of the oxy-hemoglobin concentration (Bumstead et al., 2017), with a short time lag (0.8 s) between the central and peripheral regions of the anterior circulation territory (the medial regions seem to correspond the watershed of the middle and anterior cerebral artery’s perfusion territory referring to the vascular anatomy in mice) (Xiong et al., 2017). Another awake human study investigating the 0.1 Hz hemodynamic oscillations using optical intrinsic signal imaging also showed a time lag of the signal in the frontal lobe (Rayshubskiy et al., 2014). Based on the article figures, the spatiotemporal pattern of the oscillations seems to share similar characteristics with the GMS in that the early signal is seen in the area closer to the primary motor cortex, which is found in the posterior part of the imaging field of view. However, given that the time lag in this pathological case (the entire field of view corresponds to the tumor area) seems to be much longer and that the imaging field was relatively limited, it might be difficult to conclude whether the pial arterial flow or diameter changes detected in the study correspond to the same time lag phenomenon.

While the physiological factors are also expected to affect the other arteries, the resultant changes are known to vary depending on the type of the arteries. Previous studies on the autoregulation have revealed that the cerebral blood flow velocity, as measured using transcranial doppler ultrasonography, is modulated by the arterial blood pressure fluctuations in the major cerebral arteries (Aaslid et al., 1989, 1991; Tiecks et al., 1995; Kuo et al., 1998), especially at the frequency range of 0.07–0.3 Hz in humans (Zhang et al., 1998). On the other hand, the diameter of the middle cerebral artery is known to remain practically constant during different autoregulation tests in human studies (Huber and Handa, 1967; Aaslid et al., 1991; Giller et al., 1993; Tiecks et al., 1995) in contrast to the 5–10% change reported in studies using small animals whose middle cerebral artery diameter is less than 1 mm (Kontos, 1989) or to the spontaneous diameter changes of the pial arterioles up to 50% in awake mice (Drew et al., 2011). Blood flow response to carbon dioxide is also known to differ even among the internal, external, and vertebral carotid arteries in humans (Sato et al., 2012).

Such a difference in the form of response might explain why the internal carotid artery signal is highly inversely correlated with the GMS. In the internal carotid arteries, unlike smaller cerebral arteries, changes in the mean arterial pressure are known to be inversely correlated with the diameter, while positively correlated with the blood flow velocity, resulting in a stable flow (Liu et al., 2013). The signal within the carotid artery ROI is supposed to decrease if it sufficiently constricts (Supplementary Figures 5A,B), given the present study result showing that the carotid artery demonstrates higher intensity compared with the surrounding tissues (Figure 4B). As for carbon dioxide-mediated changes, however, reports on the diameter of the internal carotid arteries are not consistent. While some showed that the internal carotid arteries dilate in response to the increase in the partial pressure of arterial carbon dioxide (Willie et al., 2012; Hoiland et al., 2016), the others found no significant change in the diameter (Sato et al., 2012; Coverdale et al., 2015). Therefore, if the carbon dioxide is the leading cause, the signal decrease in the internal carotid arteries is more likely to be caused by the intra-voxel inhomogeneity due to high flow (Supplementary Figures 5A,C). In either case, while the increase in the blood pressure or the blood flow can at least theoretically decrease the internal carotid artery signal, the simultaneous change can increase the local cerebral blood flow within a several-second delay. This will increase the BOLD signal due to the larger effect on the part of the deoxyhemoglobin decrease that increases the signal compared with the venous diameter increase that decreases the signal in the veins (Kim and Ogawa, 2012; Supplementary Figures 5D,E).

The finding that the macroscopic pattern of the BOLD signal time lag is similar for the rs-fMRI signal and the signal caused by the synchronous neural activation provides some implications for fMRI studies. Firstly, if the study aims to map the flow of neural activation, the global signal regression is not enough to eliminate the vascular effect even if it is applied, taking into account its time lag (Erdogan et al., 2016), or by using ICA to avoid introducing known biases associated with this controversial approach (Murphy et al., 2009). The resultant component still has the vascular time lag embedded in its signal and requires the HRF correction. Secondly, a simple computation of the GMS time lag can provide temporal information about the whole brain HRF. Although the precise HRF can vary even depending on the task duration (Logothetis et al., 2001; Martindale et al., 2005; Kennerley et al., 2012; Martin et al., 2013), such subject- and site-specific information would be useful to predict the BOLD response in each region for each subject to improve the sensitivity of fMRI (Handwerker et al., 2004). Finally, it is important to note that the time lag mapping of the rs-fMRI data could be flawed if not properly thresholded, as well-described in Tong et al. (2019a). The inclusion of the spuriously correlated voxels into the time lag mapping produces “another path of the traveling signal” masquerading as the flow of neural activation (as we have shown in Dataset 1, C05). Based on the highly coherent nature of the temporal properties of the BOLD response across the triggers shown in the present study, in addition to the previous study confirming a similar trend for the multiple global signals (Amemiya et al., 2019), such a phenomenon less likely to represent the flow of neural signals at least as the constant and independent components in the neural band of the human rs-fMRI data.

There are some technical considerations for the present study. Firstly, some local IC time lag map showed a relatively low correlation with that of the GMS. It might be due to a larger variance caused by the small size of the involved area. The high overall similarity between the composite maps of the local ICs and the GMS supports this view. It is also important to note that the correlation might be underestimated because we used HCP data pre-processed with sICA + FIX denoising. While the denoising strategy is beneficial in reducing motion-related artifacts, it also removes signals form the large veins and venous sinuses. Although we measured the GMS within the gray matter, given that it is generally highly correlated with the venous signals (Tong et al., 2019a), some fraction of the GMS signal must have been removed thorough the process. Secondly, while the temporal ICA is suitable in finding spatially overlapping activities, it might more likely divide one component into multiple ICs due to a time lag. However, even if it were the case, it does not affect the comparison of the time lag structures. The same also holds if the assumed origin of the local ICs were not entirely neural. Finally, although the primary source of the GMS is likely the physiological noise, it could also have some neural components. Both electroencephalographic work in humans and microelectrode recordings in anesthetized monkeys have shown widespread BOLD fluctuations to be correlated with slow changes in neural activity (Leopold et al., 2003; He et al., 2008; Scholvinck et al., 2010; Liu et al., 2018).

The neurovascular coupling is a complex phenomenon, and a comprehensive picture of the cellular and vascular mechanisms is yet to be identified (Hillman, 2014; Drew, 2019). Although our view, based on the similarity of the BOLD responses, provides some insights into the origin of the global signal and the time lag phenomenon, it would be more informative if we will be able to also address the possible difference based on the mechanism triggering the vascular response using rs-fMRI.

## Conclusion

By comparing the spatiotemporal characteristics of the rs-fMRI signal with those of the simultaneously induced neural signals, we have demonstrated that the time lag phenomenon in the rs-fMRI most likely reflects the regional variance in the hemodynamic responses rather than the time lag on the part of the stimulus or trigger and that the cause of the GMS can be substantially synchronous. The tight inverse coupling between the internal carotid artery signal and the GMS is presumably reflecting the blood flow or blood pressure changes that are occurring almost simultaneously in the internal carotid artery and the pial/capillary arteries within the 0.01–0.1 Hz low-frequency component in human rs-fMRI.

## Data Availability Statement

The raw data supporting the conclusions of this article will be made available by the authors, without undue reservation.

## Ethics Statement

The studies involving human participants were reviewed and approved by University of Tokyo. The patients/participants provided their written informed consent to participate in this study.

## Author Contributions

SA contributed to the conception and design of the study, acquired data, performed the statistical analysis, and wrote the first draft of the manuscript. All authors contributed to the final drafting of the manuscript, read and approved the submitted version.

## Conflict of Interest

The authors declare that the research was conducted in the absence of any commercial or financial relationships that could be construed as a potential conflict of interest.

## Footnotes

1 https://www.cmrr.umn.edu/multiband/
