# Supplementary material for: Origin of the Time Lag Phenomenon and the Global Signal in Resting-State fMRI
Source: Front Neurosci. 2020 Oct 29;14:596084. doi: 10.3389/fnins.2020.596084 (PMC7673396; doi:10.3389/fnins.2020.596084)
Supplement: Supplementary file 1 [file Table_1.docx]

Supplementary Material

# Supplementary Figures

**
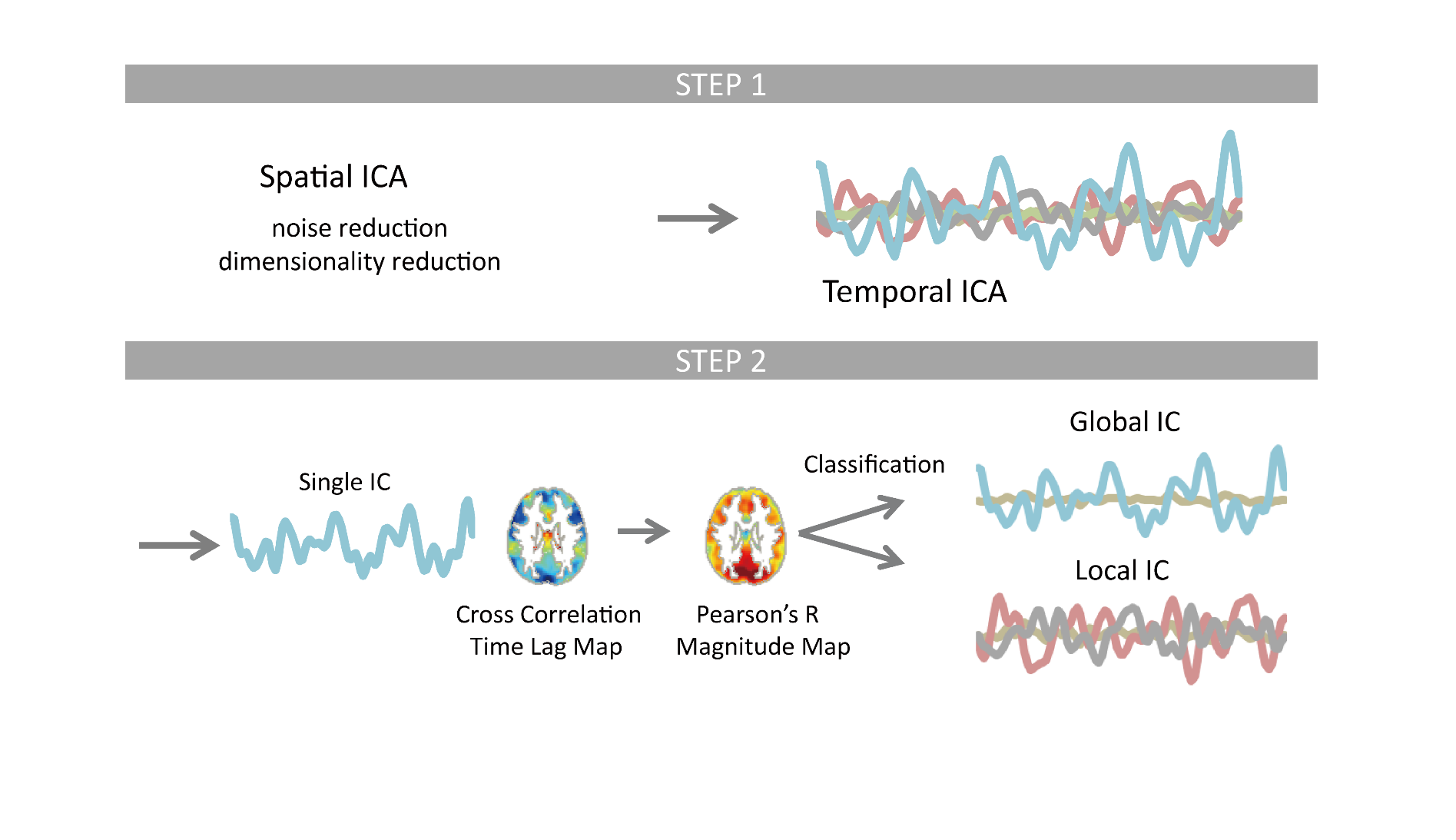
Fig. S1. Schematic of the resting-state fMRI data processing for Experiment 1.**

As described in (Amemiya et al., 2019), temporal independent component analysis (ICA) was applied to pre-processed test and re-test dataset, whose dimensionality were reduced to 61 × 120,000 and 62 × 120,000 using spatial ICA, respectively. Each temporal ICA gave 28 and 30 reproducible components (Step 1). For each component (IC time series), a time lag map was obtained by computing the time lag of each voxel relative to the IC time series using cross-correlation. Pearson’s correlation coefficients were computed between each voxel’s time series and the IC time series that was shifted as much as the measured time lag. Classification of ICs was based on spatial distribution pattern of each component. Any component that is more similar to the whole-brain signal than any RSN template in distribution pattern was classified as global (Step 2).

Fig. S2. Time lag map of the resting-state functional networks (HCP data, Dataset 2)

**
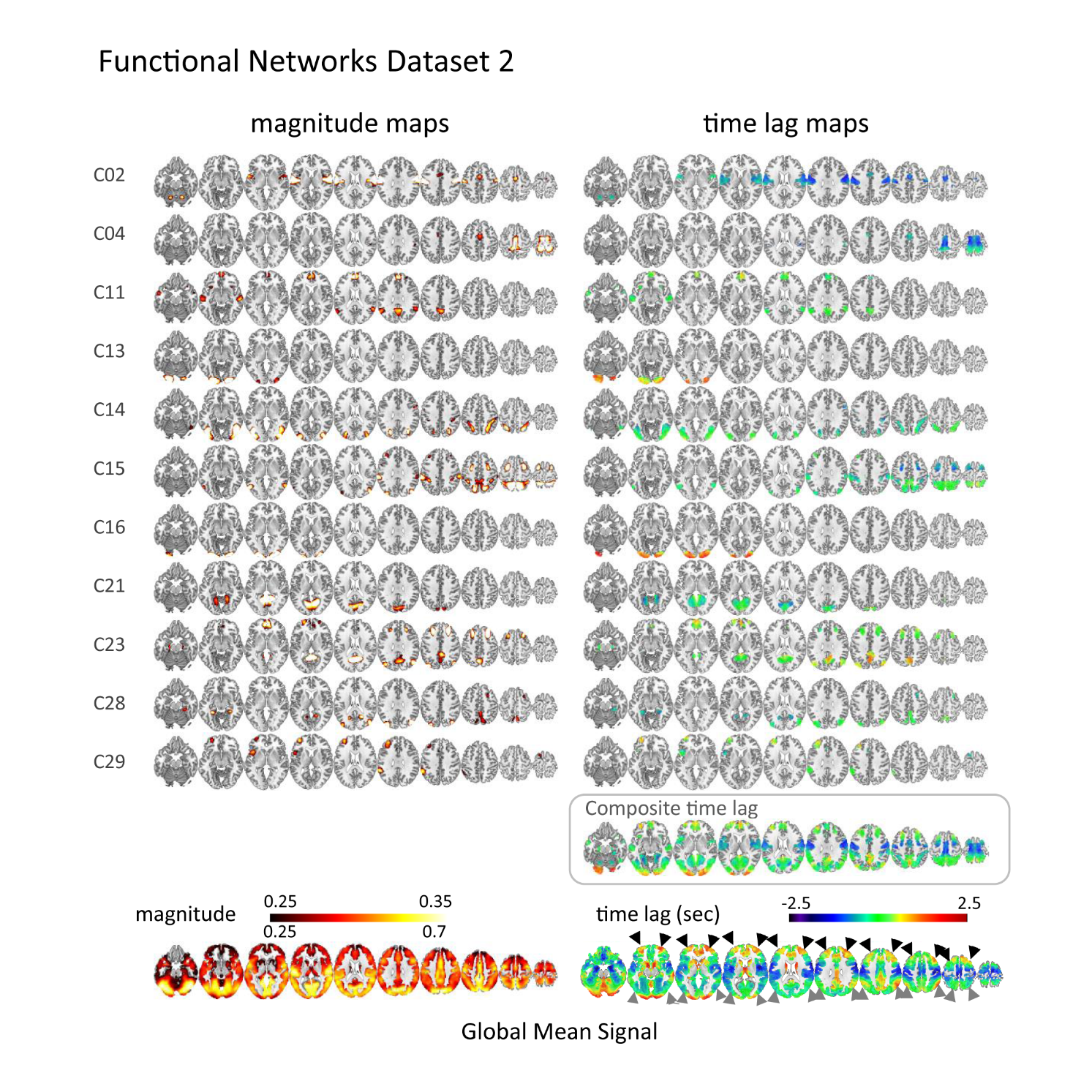
**

The magnitude and the time lag maps of the 11 ICs from Dataset 2, which was judged as a local component in the previous study (Amemiya et al., 2019) and that survived the time-series correlation threshold of Pearson’s r > 0.3 across the 100 runs as well as those of the global mean signal are shown. A larger time lag correlation was seen for the ICs with larger areas of high magnitude, such as the visual cortex (C16 and C21). The composite map incorporating all IC lag maps shows a marked similarity between the time lag maps of the local network signals and that of the global mean signal (r = 0.86, p < 2.2*10^-308^). Black and grey arrowheads in the global mean signal time lag map indicate the anterior and posterior water-shed regions, respectively.

Fig. S3. 2D histogram of the pooled time lag data (HCP data, Dataset 2)


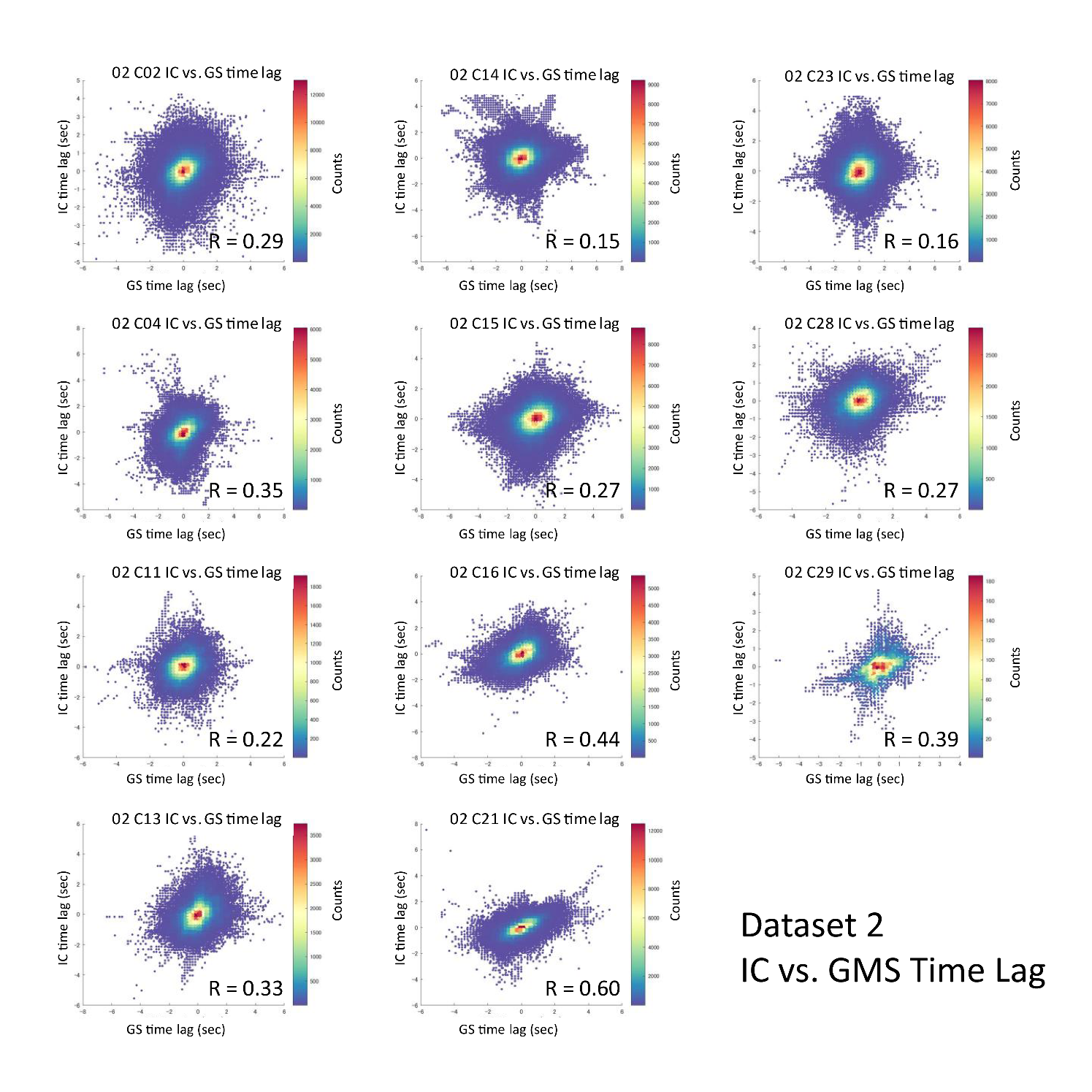


For each IC, a 2D histogram shows the relationship between the IC vs. global mean signal time lag (pooled data across subjects). All IC time lags were significantly correlated with that of the global mean signal when compared within the areas surviving the time-series correlation threshold of Pearson’s r = 0.3 (r = 0.31 ± 0.13, p < 0.001). (GMS = global mean signal)

Fig. S4. The relative time lag between the lag map of the resting-state functional networks and that of the global mean signal.


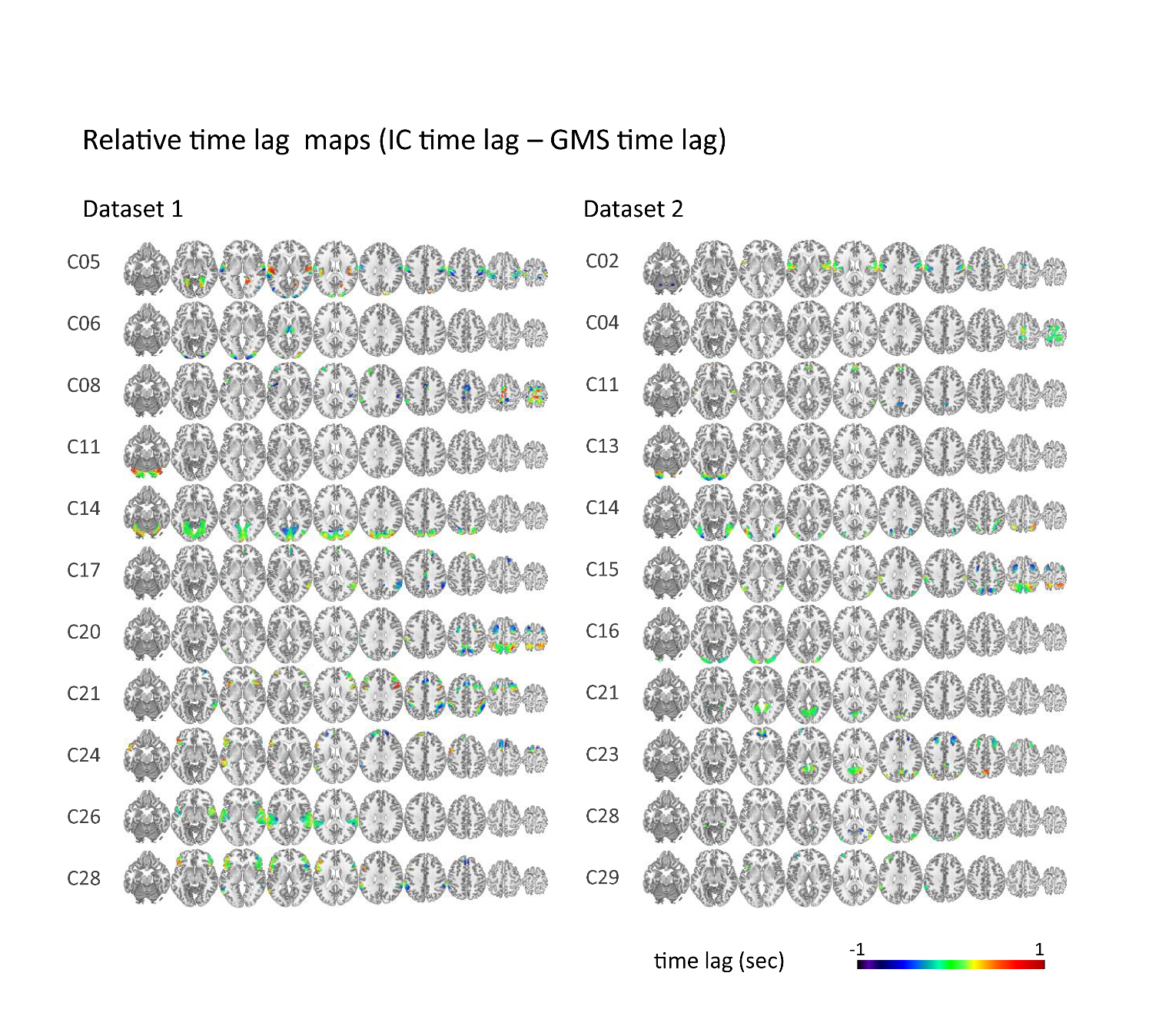


Resting-state functional networks time lag relative to that of the global mean signal was measured for each IC and averaged across the subjects. A larger time lag was more likely to be found at the periphery of the regions. (GMS = global mean signal)


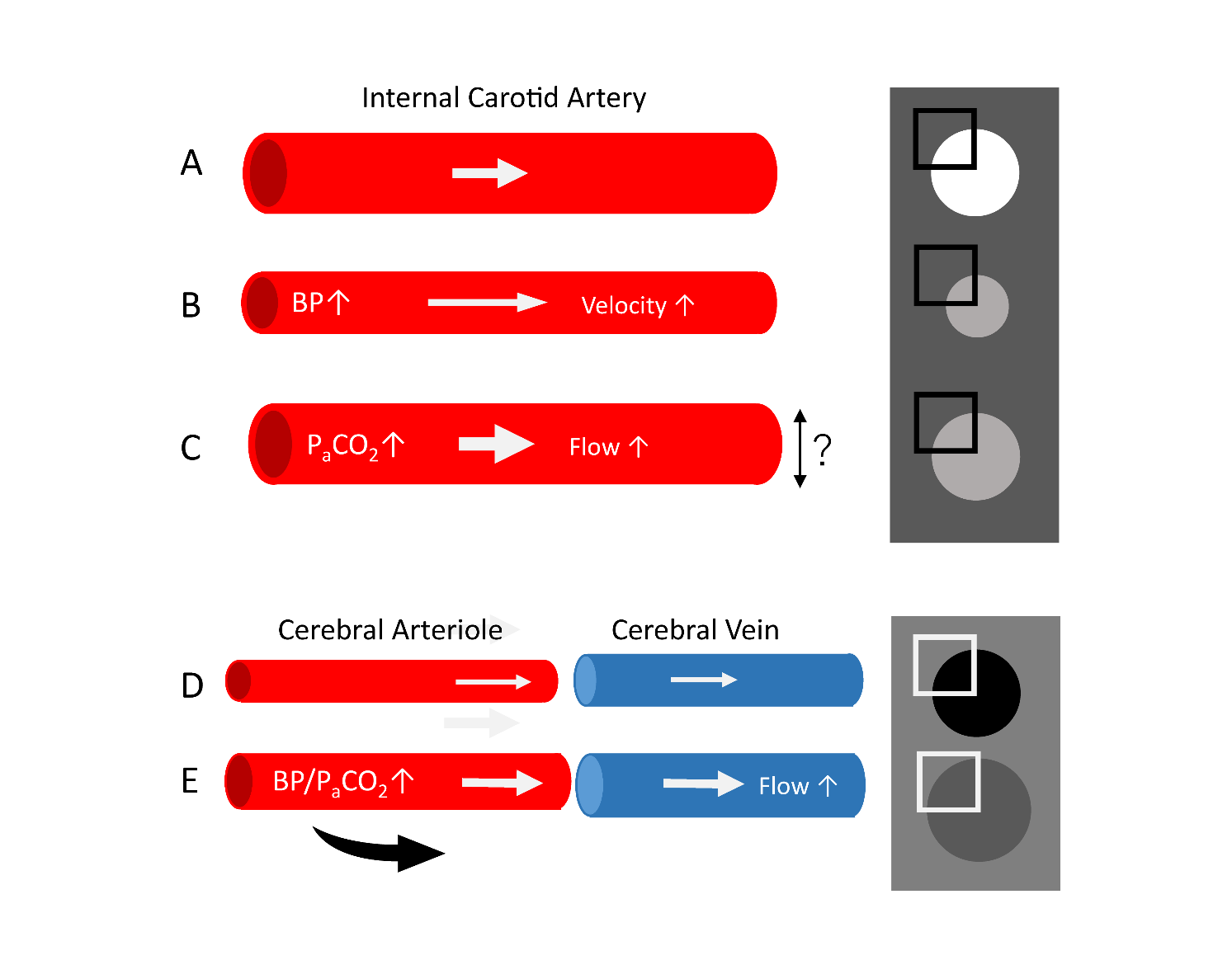
Fig. S5. Signal changes in the internal carotid artery and cerebral vein associated with the increased blood pressure and P_a_CO_2_.

In the internal carotid arteries, changes in the mean arterial blood pressure (BP) are inversely correlated with the diameter while positively correlated with the blood flow velocity, resulting in a stable flow (Liu et al., 2013) (B). The right column shows the signal intensity model of the vessels’ cross-sections (circles) relative to the surrounding tissues. The black and white boxes represent the voxels composed of both tissues. Constriction of the internal carotid artery will reduce the signal within the voxels because the internal carotid artery demonstrates higher intensity than the surrounding tissues (A vs. B). In addition to the caliber change, an increase in the velocity could cause intra-voxel inhomogeneity that also decreases the signal intensity (A vs. B). As for an increase in the partial pressure of arterial carbon dioxide (P_a_CO_2_), while it increases the internal carotid artery blood flow, it might dilate (Willie et al., 2012; Hoiland et al., 2016), or might not change the internal carotid artery diameter (Sato et al., 2012; Coverdale et al., 2015) (A vs. C). If the internal carotid artery diameter remains the same, the increase in blood flow also increases the velocity, which would reduce the signal due to intra-voxel inhomogeneity. Even if the internal carotid artery dilates, if the velocity also increases in the internal carotid arteries, it would also decrease the internal carotid artery signal (A vs. C). In the brain tissue, a similar increase in the arterial blood pressure or the P_a_CO_2_ will dilate the arterioles and increase the arterial blood flow. This is followed by an increase in the venous blood flow that dilates the local veins. As a result, the BOLD signal increases because the effect on the part of the deoxyhemoglobin decrease that increases the signal is larger than that of the venous diameter increase that decreases the signal in the veins (Kim and Ogawa, 2012) (D vs. E).

Time lag measurement of the BOLD signal using cross-correlation

If we assume that the BOLD signal is the output of a linear time-invariant system (Boynton et al., 1996), we can express the expected signal $X\left( t \right)$ as the convolution of the stimulus function $u\left( t \right)$ with a hemodynamic response function $h\left( t \right)$ as follows:

$$\begin{aligned} X\left( t \right)=u\left( t \right)\bigotimes h\left( t \right)\#\left( 1 \right) \end{aligned}$$

where $\bigotimes$ is the convolution operator.

When the stimulus time is delayed by $\Delta t$, the BOLD signal $X^{'}$ is also delayed as much as $\Delta t$, which is described as follows:

$$\begin{aligned} X^{'}(t)=u\left( t-\Delta t \right)\bigotimes h\left( t \right)=X\left( t-\Delta t \right)\#\left( 2 \right) \end{aligned}$$

The stimulus time delay $\Delta t$ can be, therefore, estimated as the BOLD signal time lag by using cross-correlation of $X$ and $X^{'}$ defined as follows:

$$\begin{aligned} \left( X*X’ \right)\left( \tau\right)\triangleq\int_{-\infty}^{\infty} \bar{X\left( t \right)}X\left( t-\Delta t+\tau\right)dt\#\left( 3 \right) \end{aligned}$$

where $*$ is the cross-correlation operator, $\tau$ is the time lag between $X$ and $X'$, and $\bar{X\left( t \right)}$ denotes the complex conjugate of $X(t)$.

By taking the square of (3), we obtain

$$\begin{aligned} \left| \left( X*X’ \right)\left( \tau\right) \right|^{2}=\left| \int_{-\infty}^{\infty} \bar{X\left( t \right)}X\left( t-\Delta t+\tau\right)dt \right|^{2}\#\left( 4 \right) \end{aligned}$$

Application of the Cauchy–Schwarz inequality to (4) gives

$$\begin{aligned} \left| \left( X*X’ \right)\left( \tau\right) \right|^{2}\leq\int_{-\infty}^{\infty} \bar{\left| X\left( t \right) \right|}^{2}dt\cdot\int_{-\infty}^{\infty} \left| X\left( t-\Delta t+\tau\right) \right|^{2}dt=\left\{ \int_{-\infty}^{\infty} \left| X\left( t \right) \right|^{2}dt \right\}^{2}\#\left( 5 \right) \end{aligned}$$

Thus, the cross-correlation is maximized when the following is true for any *t*.

$$\begin{aligned} X\left( t \right)= X\left( t-\Delta t+\tau\right)\#\left( 6 \right) \end{aligned}$$

This is equivalent to

$$\begin{aligned} \tau=\Delta t\#\left( 7 \right) \end{aligned}$$

If we suppose the hemodynamic response function to be similarly delayed by $\Delta t$, the BOLD signal is also delayed by $\Delta t$ as follows:

$$\begin{aligned} X^{'}\left( t \right)=u\left( t \right)\bigotimes h\left( t-\Delta t \right)=X\left( t-\Delta t \right)\#(8) \end{aligned}$$

Therefore, the delay $\Delta t$ is computed by using cross-correlation (3) in the same way.

References

Amemiya, S., Takao, H., and Abe, O. (2019). Global vs. Network-Specific Regulations as the Source of Intrinsic Coactivations in Resting-State Networks. *Front Syst Neurosci* 13**,** 65. doi: 10.3389/fnsys.2019.00065.

Boynton, G.M., Engel, S.A., Glover, G.H., and Heeger, D.J. (1996). Linear systems analysis of functional magnetic resonance imaging in human V1. *J Neurosci* 16(13)**,** 4207-4221. doi: 10.1523/jneurosci.16-13-04207.1996.

Coverdale, N.S., Lalande, S., Perrotta, A., and Shoemaker, J.K. (2015). Heterogeneous patterns of vasoreactivity in the middle cerebral and internal carotid arteries. *Am J Physiol Heart Circ Physiol* 308(9)**,** H1030-1038. doi: 10.1152/ajpheart.00761.2014.

Hoiland, R.L., Tymko, M.M., Bain, A.R., Wildfong, K.W., Monteleone, B., and Ainslie, P.N. (2016). Carbon dioxide-mediated vasomotion of extra-cranial cerebral arteries in humans: a role for prostaglandins? *J Physiol* 594(12)**,** 3463-3481. doi: 10.1113/jp272012.

Kim, S.G., and Ogawa, S. (2012). Biophysical and physiological origins of blood oxygenation level-dependent fMRI signals. *J Cereb Blood Flow Metab* 32(7)**,** 1188-1206. doi: 10.1038/jcbfm.2012.23.

Liu, J., Zhu, Y.S., Hill, C., Armstrong, K., Tarumi, T., Hodics, T., et al. (2013). Cerebral autoregulation of blood velocity and volumetric flow during steady-state changes in arterial pressure. *Hypertension* 62(5)**,** 973-979. doi: 10.1161/hypertensionaha.113.01867.

Sato, K., Sadamoto, T., Hirasawa, A., Oue, A., Subudhi, A.W., Miyazawa, T., et al. (2012). Differential blood flow responses to CO(2) in human internal and external carotid and vertebral arteries. *J Physiol* 590(14)**,** 3277-3290. doi: 10.1113/jphysiol.2012.230425.

Willie, C.K., Macleod, D.B., Shaw, A.D., Smith, K.J., Tzeng, Y.C., Eves, N.D., et al. (2012). Regional brain blood flow in man during acute changes in arterial blood gases. *J Physiol* 590(14)**,** 3261-3275. doi: 10.1113/jphysiol.2012.228551.
